# Regulators of ribonucleotide reductase inhibit Ty1 mobility in *saccharomyces cerevisiae*

**DOI:** 10.1186/1759-8753-1-23

**Published:** 2010-11-22

**Authors:** John P O'Donnell, Marie Gehman, Jill B Keeney

**Affiliations:** 1 Department of Biology, Juniata College, Huntingdon, PA, USA; 2 Department of Molecular Medicine, Cornell University, Ithaca, NY 14853-6401, USA; 3 University of Kentucky College of Medicine, Lexington, KY 40506, USA

## Abstract

**Background:**

Ty1 is a long terminal repeat retrotransposon of *Saccharomyces cerevisiae*, with a replication cycle similar to retrovirus replication. Structurally, Ty1 contains long terminal repeat (LTR) regions flanking the *gag *and *pol *genes that encode for the proteins that enable Ty1 mobility. Reverse transcriptase produces Ty1 complementary (c)DNA that can either be integrated back into the genome by integrase or recombined into the yeast genome through homologous recombination. The frequency of Ty1 mobility is temperature sensitive, with optimum activity occurring at 24-26°C.

**Results:**

In this study, we identified two host genes that when deleted allow for high temperature Ty1 mobility: *RFX1 *and *SML1*. The protein products of these genes are both negative regulators of the enzyme ribonucleotide reductase, a key enzyme in regulating deoxyribonucleotide triphosphate (dNTP) levels in the cell. Processing of Ty1 proteins is defective at high temperature, and processing is not improved in either *rfx1 *or *sml1 *deletion strains. Ty1 mobility at high temperature is mediated by homologous recombination of Ty1 cDNA to Ty1 elements within the yeast genome. We quantified cDNA levels in wild type, *rfx1 *and *sml1 *deletion background strains at different temperatures. Southern blot analysis demonstrated that cDNA levels were not markedly different between the wild type and mutant strains as temperatures increased, indicating that the increased Ty1 mobility is not a result of increased cDNA synthesis in the mutant strains. Homologous recombination efficiency was increased in both *rfx1 *and *sml1 *deletion strains at high temperatures; the *rfx1 *deletion strain also had heightened homologous recombination efficiency at permissive temperatures. In the presence of the dNTP reducing agent hydroxyurea at permissive temperatures, Ty1 mobility was stimulated in the wild type and *sml1 *deletion strains but not in the *rfx1 *deletion strain. Mobility frequency was greatly reduced in all strains at high temperature. Deletion of the S-phase checkpoint pathway Dun1 kinase, which inactivates Sml1 and Rfx1, reduced Ty1 mobility at a range of temperatures.

**Conclusions:**

Levels of cellular dNTPs, as regulated by components of the S-phase checkpoint pathway, are a limiting factor in homologous recombination-mediated Ty1 mobility.

## Background

Managing genome stability is a complex process, requiring a delicate balance of gene structure and sequence integrity with flexibility for genetic exchange and DNA repair. The Ty1 long terminal repeat (LTR) retrotransposons of *Saccharomyces cerevisiae *inevitably play a major role in this balance, because the yeast genome contains ~30 whole length elements and hundreds of LTR sequences. The genes *gag *and *pol *contained within Ty1 elements encode for structural proteins and enzymatic proteins, respectively. As in related retroviruses, the *gag *domain encodes for the structural protein coat of the virus-like particle (VLP) and the *pol *region consists of essential enzymatic proteins (protease, reverse transcriptase and integrase) that mediate Ty1 mobility events [1]. Ty1 elements are transcribed from the genome into mRNA which accounts for up to 0.8% of total cellular RNA [2]. The Ty mRNA is then translated and processed via a protease, which is encoded by the *pol *region of Ty1. Post processing, reverse transcriptase and integrase are active, and continue the life cycle through synthesis of complementary (c)DNA from the mRNA template and integration of the Ty cDNA into the genome. Incorporation of cDNA into the host genome can occur by *RAD52*-dependent homologous recombination (HR) or integrase-mediated integration; the term Ty1 mobility is used to describe these collective integration events.

Ty1 mobility is a temperature sensitive mechanism that occurs optimally at 20-26°C. Levels of Ty1 mobility are drastically reduced when yeast cells are grown at or above 32°C, even though cellular growth is unaffected [3]. Thus, an unknown regulatory mechanism has evolved to repress Ty1 mobility as temperature increases. We have previously analysed various steps of the Ty1 life cycle at a range of temperatures. At high temperatures (above 34°C), processing by protease of the Ty1 Gag-Pol-p199 polyprotein into structural and enzymatic protein domains is blocked, thus *gag *processing is partially inhibited and processing of integrase is completely inhibited. Additionally, reverse transcriptase activity is dampened within VLPs, and Ty1 cDNA cannot be detected by Southern blot analysis. The mechanism of Ty1 mobility shifts as the temperature increases. At permissive temperatures, the primary integration mechanism is integrase mediated; however, the relatively few high temperature mobility events are mediated by homologous recombination of Ty1 cDNA.

Ty1 mobility, regardless of mechanism, is tightly regulated by host cell factors. Ty1 mRNA is very abundant, yet transposition of endogenous Ty1 occurs at a very low level [4]. Several groups have conducted genomewide screens, identifying numerous host factors that mediate regulation of Ty1 mobility at many steps in the life cycle of the element [5-10]. To investigate the mechanism of temperature regulation of Ty1, we were specifically interested in determining the mechanism by which host cell factors limit Ty1 mobility at high temperatures. In this study, we screened a yeast deletion library for increased transposition at high temperatures (34°C). We found that deletion of *RFX1*, *SML1 or GRH1 *resulted in increased levels of Ty1 mobility at high temperatures. Interestingly, Sml1 and Rfx1 are both negative regulators of ribonucleotide reductase (RNR), which catalyzes the rate-limiting step in deoxyribonucleotide triphosphate (dNTP) synthesis. In yeast, the RNR enzyme is composed of a large homodimeric subunit originating from the *RNR1 *gene plus a small heterodimeric subunit derived from the *RNR2 *and *RNR4 *genes. *RFX1 (*also known as *CRT1) *is the predominant regulator of the *RNR2*, *RNR3 *and *RNR4 *genes. The sequence specific transcriptional repressor Rfx1 protein binds to DNA damage response elements (X boxes) located within the promoter region of the *RNR *genes, and recruits general repressors. [11-13]. The Sml1 protein regulates RNR activity by binding and inhibiting the RNR1 homodimeric subunit [14,15]. Thus, deletion of either *SML1 *or *RFX1 *results in an increase in intracellular dNTP pools [16-18]. *RFX1 *has been previously identified as a marginal regulator of Ty1 mobility [7]. In this study, we have identified *SML1 *as a novel regulator of Ty1 transposition.

**Figure 1:**
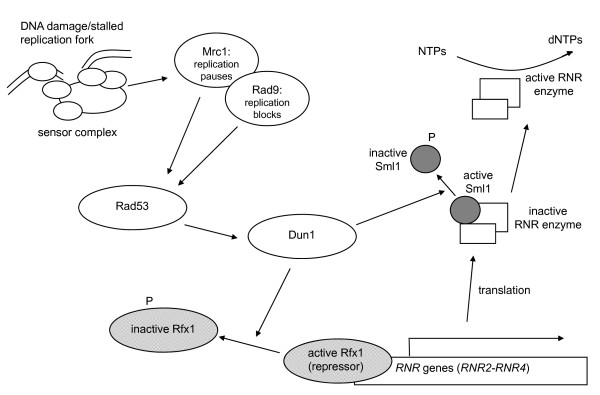
**Schematic of the ribonucleotide reductase (RNR) induction pathway**. DNA damage or stalled replication forks during S-phase activates a sensor complex that recruits Mec1 to the site. Mec1 then activates a mediator kinase; either Mrc1 at stalled replication forks, or Rad9 at damaged sites. The activated mediator kinase then activates the effector kinase Rad53, which subsequently phosphorylates Dun1. The activated Dun1 protein phosphorylates the transcriptional repressor Rfx1 (Crt1), thereby inactivating it and allowing for increased transcription of the *RNR *genes. Sml1 binds and keeps inactive the large RNR1 subunit; Dun1 phosphorylation of Sml1 causes it to release RNR1, and the enzyme becomes active. RNR converts NTPs to dNTPs as needed for DNA synthesis and repair. Only the pathway components most relevant to this study are shown.

Regulation of dNTP concentration is crucial during the S phase of the cell cycle. Low concentrations of dNTPs can lead to replication fork stalling and potential collapse, whereas high concentrations of dNTPs can result in polymerase infidelity, leading to mutations. Consequently, RNR activity is regulated at multiple levels: transcriptionally by Rfx1, post-translationally by Sml1 and allosterically by dNTPs, especially dATP [11,18,19]. Control of Sml1 and Rfx1 activity are tightly linked to S-phase and the S-phase checkpoint pathways (Figure 1). Sml1 is normally degraded during S-phase to provide dNTP substrates for DNA synthesis [20]. Additionally, activation of the S-phase checkpoint pathway in response to DNA damage or stalled replication forks leads to an increase in RNR activity [20]. Activation of the S-phase checkpoint begins with binding of the sensor complex to affected DNA in response to either replication blocks or pauses. The sensor complex recruits the essential kinase Mec1. A mediator kinase (either Mrc1 for replication pauses or Rad9 for replication blocks) is then phosphorylated, which subsequently activates the effector kinase Rad53. [21-27]. The activated Rad53 kinase activates Dun1, which in turn phosphorylates and subsequently inactivates Rfx1 and Sml1. [11,12,18,28,29]. The resulting increase in *RNR *gene transcription and active RNR protein provides the increased levels of dNTPs necessary to a cell dealing with replication problems [30].

Several host factors linking Ty1 mobility to S-phase checkpoint pathways and genome stability have been identified [7,10]. Recently, Curcio *et al*. characterized mutations in a set of repressor of Ty1 transposition (*rtt*) genes [31]. They showed that mutations in S-phase checkpoint pathways increase Ty1 mobility in 19 *rtt *mutants involved in genome stability. These *rtt *mutations play a role in the S-phase Rad53 effector pathway, separate from dNTP regulation. Although mobility of Ty1 elements does not require S-phase checkpoint proteins, the observation that Ty1 activation is mediated by an increase in Ty1 protein levels and reverse transcriptase activity in *rtt101 *mutants suggests that Ty1 mobility is an integral part of the DNA damage response [31,32]. Indeed, Ty1 has been shown to play a role in repair of chromosomal lesions [32,33].

To understand how increased dNTP levels in *sml1Δ *and *rfx1Δ *(deletion) mutants relate to increased Ty1 mobility, we investigated multiple steps within the Ty lifecycle. We quantified transposition and cDNA levels in wild type, *rfx1Δ and sml1Δ *strains at varying temperatures. Southern blot analysis demonstrated that cDNA levels did not differ between the wild type and mutant strains as temperature increases, indicating that increased Ty1 mobility is not a result of increased cDNA synthesis in the deletion strains. Homologous recombination efficiency was increased in both *rfx1Δ *and *sml1Δ *strains at high temperatures, suggesting that the increased mobility results from increased homologous recombination of Ty1 cDNA. Mobility assays were also conducted in the presence of the dNTP reducing agent, hydroxyurea (HU), which eliminated the high temperature phenotype in both *rfx1Δ *and *sml1Δ *strains. Our results show that dNTP concentrations are a limiting resource for Ty1 mobility at high temperatures.

## Results

### Genetic screen for regulators of high temperature transposition

Ty1 transposition is temperature sensitive, mainly due to inactivity of the protease enzyme and reduction of protein levels at high temperatures [3]. To study specific host regulation of transposition, we screened a yeast deletion library for mutants that transpose under high temperature conditions of 34°C [34]. An aliquot of the *MATa *deletion pool was transformed with a galactose-inducible Ty1 element (pGTy1) marked with *his3AI *on plasmid pGTy1H3m*his3AI *[35]. With this plasmid, transposition is assayed by plating cells onto a medium containing galactose to induce transposition, followed by plating to a medium lacking histidine to select for Ty1 mobility. Upon splicing and subsequent reverse transcription of the *his3AI *marker gene, a functional *HIS3 *gene sequence can be incorporated into the yeast genome, either by integration via the Ty1 integrase or by recombination by the host cellular machinery. The resulting His-positive papillae are collectively referred to as pGTy1 mobility events, because this assay cannot distinguish between integration and recombination. pGTy1H3m*his3AI *transformants of a *MATa *deletion pool were induced on galactose medium at 34°C and subsequently screened for growth on synthetic complete medium lacking histidine (SC-His) at levels higher than background colonies. Putative high temperature clones were then screened in a secondary patch assay. Nine final clones were isolated, and the deleted open reading frames (ORFs) were identified by PCR and sequencing. The nine final clones included three each with deletions of the genes *SML1*, *RFX1/CRT1 *and *GRH1*. Rfx1 and Sml1 are both involved in regulation of the RNR enzyme, which converts NTPs to dNTPs for DNA synthesis and repair. Grh1 is a *cis*-Golgi localized protein involved in endoplasmic reticulum to Golgi transport[36]. This study focused on the role of Sml1 and Rfx1 in Ty1 mobility.

**Figure 2:**
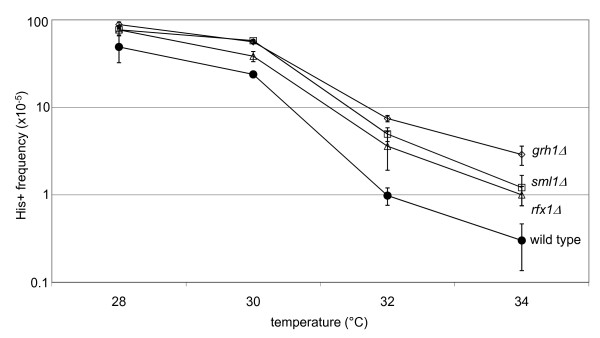
**Deletion mutants show increased Ty1 mobility at high temperature**. Isogenic wild type and mutant deletion strains were assayed for pGTy1 mobility at 28, 30, 32 and 34°C. Black circles indicate wild type (JKc1356), open squares are *sml1Δ *(JKc1357); open triangles are *rfx1Δ *(JKc1358); open diamonds are *grh1Δ *(JKc1359). The mutant strains show higher levels of pGTy1 mobility compared with wild type. Each point represents the average of three measurements; error bars indicate the standard deviation. See Additional file 1 for numerical values.

The transposition level of each deletion strain was assayed using a quantitative mobility assay (Figure 2; Additional file 1). There was a slight increase in mobility in the mutant strains at permissive temperatures. As the assay temperature increased, mobility decreased in all strains, but dropped faster in the wild type strain. The difference in pGTy1 mobility in the mutant strains was two-fold to 10-fold above wild type at 34°C.

### The high temperature mobility phenotype is strain specific and is not additive

The yeast deletion mutant strains have been shown to contain secondary mutations that affect phenotypic screens [37]. To ensure that the high temperature phenotype was a direct effect of the originally deleted mutation, fresh *LEU2 *deletions were constructed for each mutation in yeast strain Hansen BY4741. These strains were transformed with plasmid pGTy1*his3*AI[*Δ*1] and assayed for transposition; these strains maintained the high temperature transposition phenotype (data not shown). We also wanted to test whether double deletion strains showed a stronger high temperature transposition phenotype. Pairwise combinations of genes disrupted with the *kanmx4 *or *LEU2 *marker genes were constructed, transformed with pGTy1*his3*AI[*Δ*1], and assayed for pGTy1 mobility at permissive and high temperatures (34°C). We found that although the level of transposition in the double mutants always matched the phenotype of the individual mutation with the strongest high temperature phenotype, the double deletion strains were not additive (data not shown). This is not unexpected given the precise regulation of RNR activity and the inhibition of cell cycle progression by constitutively high dNTP levels [19,38]. There is likely to be a carefully regulated threshold level of dNTPs, so that removal of one or two regulatory measures is compensated for by other means.

We also constructed deletion mutations for each gene in yeast strain JKc1011, which originates from the GRF167 background. This strain shows consistently higher levels of transposition as compared with the yeast deletion strain Hansen BY4741 [8]. In patch assays, the GRF167-derived deletion strains did not demonstrate a high temperature mobility phenotype as compared with the wild type strain (data not shown). Thus, the temperature effect of these deletion mutations is strain specific.

### Protein processing not affected in mutant strains

Processing of Ty1 translation products by the Ty1 encoded protease is an ordered process, essential for efficient transposition of the Ty1 element [39]. The primary Gag translation product is cleaved to produce the capsid protein, the structural component of VLPs. The Gag-Pol translation product is cleaved by protease into capsid, protease, integrase and reverse transcriptase. We previously showed that protease processing of the Gag-Pol polyprotein is defective at high temperatures [3]. Thus, we investigated processing of the Ty1 Gag and Pol proteins in wild type and the *rfx1Δ *and *sml1Δ *mutant strains. Strains containing the pGTy1*his3*AI[*Δ*1] plasmid were induced by galactose in liquid medium at permissive (26°C) and high (34°C) temperatures; extracts of protein from aliquots removed at 0, 6 and 12 hours of induction were separated by SDS-PAGE and immunoblotted using a polyclonal antiserum to Gag or a monoclonal antibody to Pol. A polyclonal serum to glyceraldehyde-3-phosphate dehydrogenase (Gadph) was used as a loading control.

**Figure 3:**
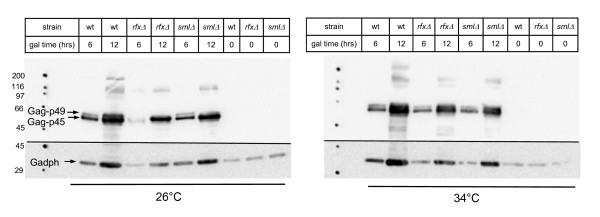
**Gag protein processing not affected in mutant strains**. Yeast strains containing the galactose-inducible plasmid pGTy1*his3*AI[*Δ*1] were grown in galactose containing medium at 26°C (left panel) or 34°C (right panel) for the indicated time. Total cell extracts were separated by SDS-PAGE and immunoblotted for Ty1 gag protein or control protein Gadph. Unprocessed Gag-p49 and processed Gag-p45 were detectable in all strains after 6 or 12 hours of galactose induction at both 26°C (left) and 34°C (right). Molecular weight markers (in kDa) are shown on the left. Strains are wild type JKc1356; *rfx1Δ *JKc1427; *sml1Δ *JKc1429.

The Gag antiserum detects the primary Gag-p49 translation product and the subsequent processed Gag-p45 capsid product. No Gag protein products were detectable before galactose induction (Figure 3). Gag-p49 and Gag-p45 were both detectable after 6 hours of galactose induction in all strains at 26°C (Figure 3, left) and 34°C (Figure 3, right). The ratio of Gag-p49 to the processed Gag-p45 product did not vary with strain genotype or temperature after 6 hours of induction. Thus, it is unlikely that the deletion mutants affect processing of the Gag protein.

**Figure 4:**
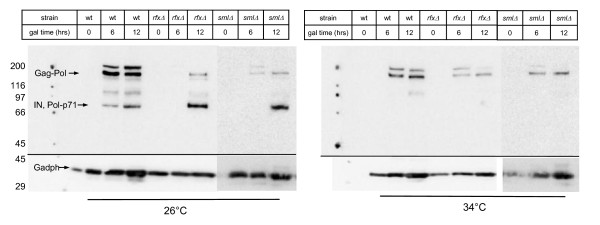
**Pol protein processing is not affected in mutant strains**. Indicated yeast strains containing the galactose-inducible plasmid pGTy1*his3*AI[*Δ*1] were grown at 26°C or 34°C in galactose for 0, 6 or 12 hours. Cell extracts were separated on SDS-PAGE and immunoblotted with monoclonal antibody to integrase or Gadph as a loading control. The integrase product was detected at 26°C only. Although the *rfx1 *and *sml1 *deletion strains showed more efficient processing of integrase at 26°C, no processed integrase was detected in any strains at 34°C. Molecular weight markers (in kDa) are shown on the left. Strains are wild type JKc1356; *rfx1Δ *JKc1427; *sml1Δ *JKc1429.

The monoclonal antibody 8B-11 detects the primary, unprocessed Gag-Pol-p199 polyprotein, several processing intermediates, and the processed 71 kD integrase (pol-p71) (Figure 4). The processed integrase was undetectable in extracts from cells induced at high temperature (Figure 4, right) consistent with our previous data Although processing of the integrase protein at 26°C appeared to be more efficient in both the *rfx1Δ *and *sml1Δ *deletion strains compared with wild type (Figure 4, left), neither of the mutant strains had detectable processed integrase at 34°C (Figure 4, right). Thus, the mutant strains have no detectable effect on the processing of the Ty1 Gag-Pol-p199 at high temperatures.

### Dependence of high temperature mobility phenotype on recombination

**Figure 5:**
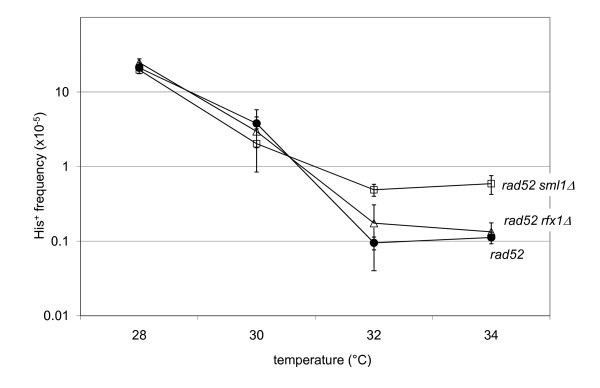
**Dependence of high temperature phenotype on recombination**. A *rad52 *mutation was introduced into wild type and each of the mutant strains. pGTy1 mobility was then determined for each strain. Black circles indicate *rad52 *(JKc1363); open squares are *rad52 sml1Δ *(JKc1361); open triangles *rad52 rfx1Δ *(JKc1360). The *rad52 *mutation eliminates the high temperature (34°C) mobility in the *rfx1 *deletion strain, but not in the *sml1 *deletion strain. Each point represents the average of three measurements; error bars indicate the standard deviation. See Additional file 2 for numerical values.

As described above, Ty1 mobility refers to integration or recombination events. We have shown previously that recombination events represent a fraction of events at permissive temperatures and that the fraction of events resulting from recombination increases as the temperature increases [8]. Recombination-mediated and integration-mediated transposition events can be distinguished by assaying for Ty1 mobility in the absence of *RAD52*, which is required for homologous recombination [40]. Thus, mobility assays conducted in strains containing a *rad52 *mutation will only yield integrase-mediated events. We constructed *rad52 *deletions in each of the mutant strains and quantified pGTy1 mobility levels. The high temperature (34°C) phenotype of the *rfx1Δ *strain was *RAD52 *dependent (Figure 5; Additional file 2), indicating that the high temperature pGTy1 mobility in this strain occurred via recombination. However, the increased pGTy1 mobility seen in the *sml1Δ *strain at 32 and 34°C appeared to be due to integrase-mediated transposition events, as the difference in mobility between the *rad52 *and *rad52 sml1Δ *strains was similar to the difference in mobility between the wild type and *sml1Δ *strain.

### Ty1 cDNA levels are not increased in *rfx1Δ *and *sml1Δ *mutants

In the *rfx1Δ *and *sml1Δ *mutants, the high temperature phenotype is probably primarily due to recombination, and processing of the Gag-Pol protein remains defective. These results suggest that the increase in Ty1 mobility at high temperatures in *rfx1Δ *and *sml1Δ *mutants is due to increased recombination of Ty1 cDNA. It is plausible that the increase in intracellular dNTPs could stimulate reverse transcriptase to produce relatively more Ty1 cDNA or that the efficiency of homologous recombination is increased.

**Figure 6:**
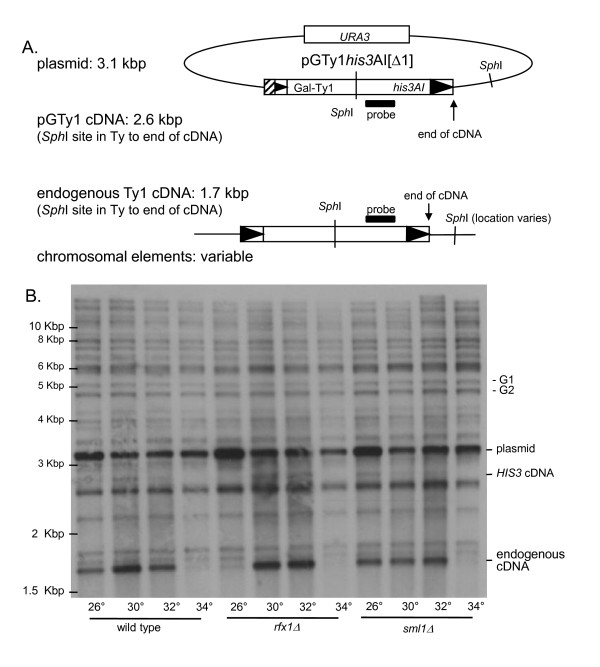
**Ty1 complementary (c)DNA levels in wild type and mutant yeast strains**. Total genomic DNA was isolated from each strain, digested with *Sph*I, transferred to nitrocellulose and probed for *Pol *sequences. **(A) **Diagram and expected size (in kbp) of Ty1 sequences detected by the *Pol *probe (modified with permission [31]). *Sph*I sites are indicated by thin vertical lines and regions hybridizing to the *Pol *probe are shown as black rectangles. Ty1 LTR sequences are represented as black triangles; the hatched box on the plasmid represents the galactose-inducible promoter.**(B) **Autoradiogram of the Southern blot. Strains and temperature of galactose induction (°C) are indicated below the blot. The locations of the pGTy1 and Ty1 sequences detected by the *Pol *probe are indicated on the right; the locations of molecular size standards are indicated on the left. The endogenous Ty1 cDNA signal in each lane was normalized to the genomic bands G1 and G2. The ratio of cDNA signal relative to 30°C within each strain and the ratio of cDNA relative to wild type between strains is given in table 1. Strains are as follows: wild type JKc1356; *rfx1Δ *JKc1427; *sml1Δ *JKc1429.

To test the first possibility, we compared Ty1 cDNA levels in wild type and deletion strains by hybridizing *Sph*I-digested DNA with a probe to the Ty1 Pol gene region (Figure 6A) [7]. This probe hybridized to both plasmid and genomic sequences and resulting cDNAs (Figure 6A). The pGTy1his3AI[Δ1]Ty1 plasmid, which is present in high copy, was detected as a prominent band at 3.1 kbp. pGTy1 cDNA from galactose induction of cells containing the plasmid was detected as a faint band at 2.6 kbp. Endogenous Cellular recombination efficiency in wild type and deletion strains.

Ty1 cDNA was the lowest molecular weight band on the Southern blot, at 1.7 kbp. The probe also hybridized to molecular weight fragments of varying sizes derived from genomic Ty1 elements. The 2.6 kbp pGTy1-derived cDNA, although faint, appeared to mirror the expression pattern of the 1.7 kbp Ty1 cDNA. The endogenous cDNA was readily visible in all strains at 30°C, but barely visible at 34°C.

Endogenous Ty1 cDNA levels were quantified by normalizing to genomic Ty bands (Figure 6, G1 and G2). The relative change in cDNA with temperature variation was determined by normalizing to the signal at 30°C within each strain (Table 1). As expected, as temperature increased, the level of cDNA dropped; at 34°C the level of cDNA was reduced to about one-tenth of the levels at 30°C. We also compared cDNA levels between strains at each temperature by normalizing to wild type cDNA levels (Table 1). No difference in cDNA level was seen between the wild type and deletion strains at each temperature, with the notable exception of the decreased level of cDNA in the *rfx1Δ *strain at 26°C. Thus, the increase in pGTy1 mobility at high temperature is not due to an increase in cDNA levels in the *rfx1Δ *and *sml1Δ *strains.

**Table 1:** Relative cDNA levels in wild type and mutant strains

	Wild type				*rfx1Δ*				*smlΔ*			
Temperature, °C	26	30	32	34	26	30	32	34	26	30	32	34

Fraction of 30°C	0.66	1	0.72	0.09	0.12	1	1.14	0.1	0.51	1	0.78	0.07

Fraction of wild type	1	1	1	1	0.16	0.88	1.4	0.95	1.09	1.41	1.53	1.02

The dramatic decrease in Ty1 cDNA in the *rfx1Δ *mutant at 26°C is unexpected. (Figure 6). It should be noted that for detection of endogenous cDNA, strains are routinely grown at 20°C [31]. However, in *rfx1Δ *strains grown at 20°C, endogenous cDNA was undetectable by Southern blot analysis (data not shown). The reduction of Ty1 cDNA in the *rfx1Δ *strain at low temperatures indicates that the *rfx1Δ *strain may be cold sensitive [41]. We assayed transposition in the *rfx1Δ *mutant at cold temperatures and found that transposition was only slightly reduced compared with wild type at 15°C, and was identical to wild type at 20°C (data not shown). We conclude that very low levels of cDNA, although undetectable by Southern blot analysis, are sufficient to yield measureable pGTy1 mobility in a patch assay.

### Cellular recombination efficiency in wild type and deletion strains

**Figure 7:**
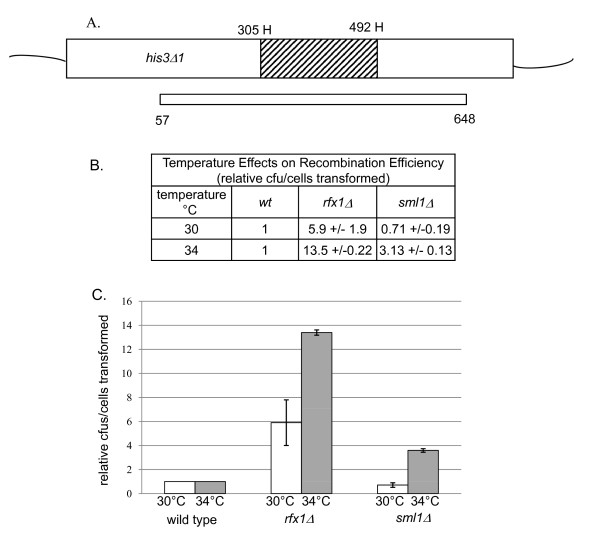
**Homologous recombination efficiency in wild type and mutant strains. (A) **Schematic of the homologous recombination assay. A PCR product spanning the deleted region of the genomic *his3Δ1 *allele was transformed into competent yeast cells. Homologous recombination of the PCR fragment with the genomic allele generates His-positive colonies. The hatched box indicated the *Hin*dIII (H) fragment deleted in the *his3Δ1 *allele. Numbers refer to base pair locations within the *HIS3 *open reading frame. **(B) **Recombination rate, relative to wild type, was measured as the efficiency of His-positive prototroph formation (cfus/cells transformed) at 30 or 34°C. The numbers represent the average of three transformation reactions and the standard deviation is given for each value. Efficiency of transformation was normalized to the transformation efficiency of a *HIS3 *marked 2κ plasmid compared with wild type. **(C) **Graphical representation of numbers given in (B). Strains are as follows: wild type JKc1046; *rfx1Δ *JKc1532; *sml1Δ *JKc1533.

Ty1 cDNA levels are not increased in *sml1Δ *and *rfx1Δ *mutant strains at high temperatures. Thus, we hypothesized that if increased cellular dNTPs in the deletion mutants mimic the DNA repair response, homologous recombination activity may be increased in these strains, thus the available Ty1 cDNA would be more efficiently recombined in the mutant strains, resulting in increased activity in the Ty1 mobility assay. We assayed cellular recombination using a modified gap-repair assay [8]. A PCR product spanning the deleted region of the *his3Δ1 *allele was transformed into competent yeast cells that were grown and transformed at 30 and 34°C. Repair of the *his3Δ1 *deletion by homologous recombination to the PCR product results in formation of histidine prototrophs, and the homologous recombination efficiency is calculated as the number of histidine prototrophs per cells transformed. This ratio is then multiplied by a transformation efficiency factor determined by transforming a *HIS3 *2-micron plasmid. A schematic of the assay and the results are shown in Figure 7. At 30°C, homologous recombination in the *rfx1Δ *mutant was increased by more than five-fold compared with wild type, and homologous recombination in the *sml1Δ *mutant was comparable with wild type. At 34°C, both deletion strains had an increase in recombination compared with the wild type. The *rfx1Δ *deletion strain increased by 13-fold and the *sml1 *strain by around threefold (Figure 7B, C). Transformation efficiency was adjusted for cell number. The transformation efficiency of the 2-micron plasmid was reduced in *rfx1Δ *cells, especially at 30°C. The reason for this is unknown.

### Effects of HU on transposition

**Figure 8:**
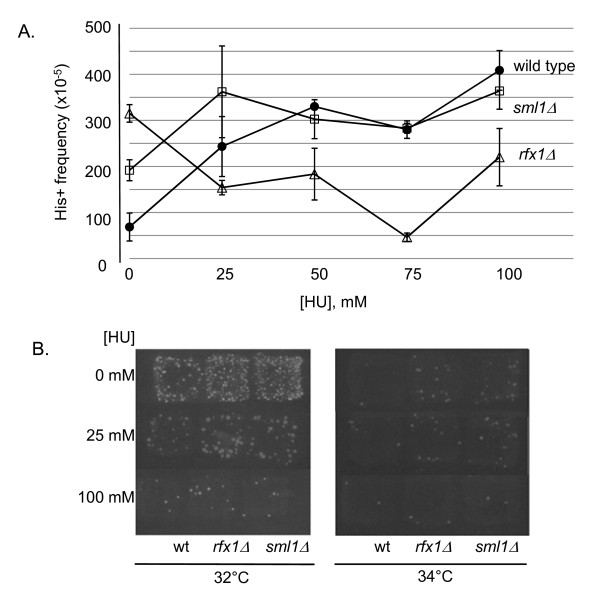
**Effect of hydroxyurea on Ty1 mobility. (A) **Yeast strains containing the galactose inducible Ty1 element on plasmid pGTy1*his3*AI[*Δ*1] were induced at permissive temperature (28°C) in the presence of varying concentrations of HU and quantitatively assayed for pGTy1 mobility. HU treatment increases pGTy1 mobility in both wild type and *sml1Δ *strains, but not in the *rfx1Δ *strain. **(B) **pGTy1 mobility patch assay at 32 and 34°C. HU treatment drastically reduces pGTy1 mobility in all strains at elevated temperatures. See Additional file 3 for numerical values.

We propose that the increase in dNTP levels in *rfx1Δ *and *sml1Δ *strains increases the HR efficiency of Ty1 cDNA, resulting in increased pGTy1 mobility at high temperature. Thus, we predict that pGTy1 mobility at high temperature would decrease if dNTPs are limited. We used the RNR inhibitor HU to decrease the cellular levels of dNTPs. In yeast, HU impedes DNA synthesis and limits the expansion of dNTP pools above basal levels during G1/S phase [42]. Interestingly, it has previously been shown that treatment with HU elevates Ty1 cDNA levels and endogenous Ty1 mobility at permissive temperatures [31]. Wild type, *rfx1Δ *and *sml1Δ *deletion strains containing plasmid pGTy1*his3*AI[*Δ*1] were induced in galactose medium without or with HU. We found that in the presence of HU, pGTy1 mobility at 28°C (permissive temperature) increased modestly (two to three times) in both the wild type and *sml1Δ *strains, but there was no induction of pGTy1 mobility in the *rfx1Δ *strain (Figure 8A; Additional file 3). pGTy1 mobility in the presence of HU at high temperature was measured using a patch assay. pGTy1 mobility decreased with increasing concentration of HU, and the presence of HU eliminated the high temperature transposition phenotype in both *sml1Δ *and *rfx1Δ *strains (Figure 8B). Attempts to quantify transposition at 34°C (high temperature) in the presence of HU did not yield adequate His-positive events to be analyzed quantitatively. This result supports the conclusion that dNTP concentrations are a limiting resource for pGTy1 mobility at high temperatures.

### Effects of DUN1 on Ty1 mobility

We wondered whether altering the activity of proteins upstream in the S-phase checkpoint pathway would have an effect on Ty1 mobility. Phosphorylation of Sml1 and Rfx1 by Dun1 releases the repressive effects of these regulators (Figure 1). Thus, overexpression of Dun1 might be expected to increase RNR activity through inactivation of Rfx1 and Sml1, resulting in increased dNTP pools and a concomitant increase in Ty1 mobility at high temperature. Conversely, deletion of Dun1 would mean that Sml1 and Rfx1 would retain their suppressive activity, so that RNR is not activated. Thus, we would expect a decrease in cellular dNTP pools, resulting in a decrease in the Ty1 high temperature mobility phenotype.

**Figure 9:**
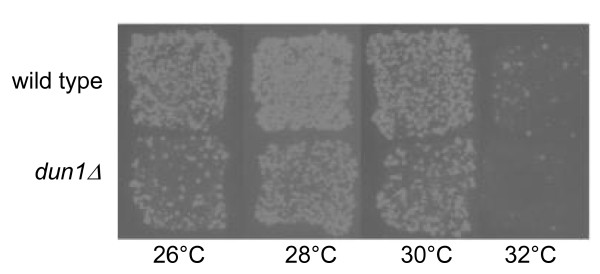
**Effect of DUN1 on high temperature transposition**. pGTy1 mobility was assayed in a *dun1 *deletion strain (JKc1445) by detection of His-positive prototrophs in a patch assay. pGTy1 mobility is decreased compared with wild type at all temperatures assayed.

A high copy *LEU2 *2 μ plasmid containing the *DUN1 *gene was transformed into a yeast strain containing the galactose inducible plasmid pGTy1*his3*AI[*Δ*1]. Transformants were then assayed for transposition at 32 and 34°C. Overexpression of Dun1 had no effect on pGTy1 mobility (data not shown). The plasmid pGTy1*his3*AI[*Δ*1] was also transformed into a *dun1Δ *strain and assayed for transposition at 26, 28, 30 and 32°C using a patch assay. As predicted, deletion of Dun1 decreased pGTy1 mobility compared with wild type across the range of temperatures (Figure 9), which is consistent with an integration of the S-phase checkpoint pathway into regulation of Ty1 mobility.

## Discussion

Retroelements are regulated by hundreds of host proteins that integrate into numerous cellular pathways [9]. A curious aspect of Ty1 regulation is the temperature sensitivity of the retro-element; Ty1 mobility decreases rapidly with increasing temperature, even though cell growth is not impaired. We have shown previously that a defect in Ty1 protease at high temperatures (34-35°C) results in a lack of processed integrase and a reduction in reverse transcriptase activity [3]. As a result, the mechanism of Ty1 mobility shifts from integrase-mediated to HR-mediated as temperature increases [8]. Correspondingly, it has been shown that Ty1 cDNA is used for homologous recombination at permissive temperatures when integrase function is blocked [40]. In this study, we found that deletion of either of the RNR negative regulators, Sml1 or Rfx1, increased pGTy1 mobility, especially at high temperatures. Our results show that the pGTy1 mobility increase in *rfx1Δ *and *sml1Δ *mutants at 34°C is due to an increase in cellular HR efficiency, probably mediated by an increase in cellular dNTP levels. Importantly, other studies have quantified dNTP levels in yeast, and have shown a twofold to fivefold increase of dNTP concentrations in *rfx1Δ *and *sml1Δ *strains [16,17].

### Increased Ty1 mobility mediated by increased homologous recombination, not Ty1 protein activity

Ty1 mobility can be regulated at multiple steps of the retroviral lifecycle, including protein processing, cDNA production and cDNA integration. We investigated each of these steps and determined that the increase in pGTy1 mobility in *rfx1 *and *sml1 *deletion strains is due to increased efficiency in HR of Ty1 cDNA. We previously showed that Ty1 protein processing is less efficient at high temperatures [3]. Thus, we investigated protein processing of *gag *and *pol *proteins in the *rfx1Δ *and *sml1Δ *strains. Although processing of the 71 kDa integrase protein was more efficient at 26°C in the deletion strains, none of the strains have detectable 71 kDa integrase at 34°C; the processing of Gag did not differ between wild type and mutant strains (Figure 3, Figure 4). Thus, the increase in pGTy1 mobility in *rfx1 *and *sml1 *deletion strains at high temperature is not the result of an increase in Ty1 protein expression or processing.

It is possible, however, that the modest increase in pGTy1 mobility at 28 to 30°C in the mutant strains (Figure 2) could be mediated by more efficient processing of integrase (Figure 4). Deletion of the *RTT101 *gene (a cullin involved in replication through damaged DNA and pause sites) increased mobility of an endogenous Ty1 element, and increased levels of reverse transcriptase and integrase proteins [31]. It is important to note, however, that a plasmid encoded galactose-inducible Ty1 (pGTy1) element is required for protein studies and the frequency of mobility from a pGTy1 element is not increased in an *rtt101Δ *strain. Thus, it is thought that mobility of pGTy1 elements is limited at a late step, perhaps integration. Because we did not see an increase in cDNA levels in either deletion strain (discussed below), it is just as plausible that the modest increase in pGTy1 mobility at permissive temperatures in the deletion strains is the result of increased HR rather than an increase in processed integrase. Indeed, there was no difference in mobility between the wild type and mutant strains in a *rad52 *background at permissive temperature (Figure 5).

Our original working hypothesis was that the increase in pGTy1 mobility at high temperatures in *rfx1Δ *and *sml1Δ *mutants was due to an increase in Ty1 cDNA resulting from increased dNTP substrates for reverse transcriptase. In fact, retroviral reverse transcriptase activity has been shown to vary with dNTP concentration. Oncoretroviruses and retroviruses show an increase of replication during cell division, when dNTP levels are elevated. Lentiviruses, such as HIV-1, are also capable of efficiently replicating in non-dividing cells (such as terminally differentiated cells) that have lower dNTP concentrations, because lentiviral reverse transcriptase has evolved to synthesize cDNA efficiently in these conditions [43]. We investigated the levels of Ty1 cDNA by Southern blot analysis using a restriction enzyme that would differentiate between Ty1 plasmid DNA, chromosomal Ty1 elements and their respective cDNAs (Figure 6A). We expected to see an increase in endogenous Ty1 cDNA and plasmid-derived cDNA in the deletion strains compared with the isogenic wild type strain. However, the analysis found no detectable difference in endogenous cDNA between strains as the temperature increased (Figure 6B). Thus, the increase in pGTy1 mobility at high temperature is probably not the result of increased Ty1 cDNA.

An interesting result of the Southern blot analysis was the marked reduction of endogenous cDNA in the *rfx1Δ *strain grown at 26°C (Figure 6, table 1). This was particularly unexpected because *RFX1 *was previously reported by Scholes *et al*. as a marginal regulator of Ty1 cDNA-mediated mobility. That group found Ty1 cDNA to be modestly increased in the *rfx1 *mutant, with the relative cDNA level measured at 1.7 compared with the parent strain [7]. However, there are several potentially important differences between their study and the present one. Scholes *et al*. measured mobility of an endogenous Ty1 element in a GRF167-derived strain. In this study, we measured pGTy1 mobility in a Hansen-derived strain. We showed previously that pGTy1 mobility levels are considerably higher in GRF167-derived strains at 26°C, perhaps due to more efficient production of Ty1 cDNA [3]. Additionally, Scholes *et al*. characterized an *rfx1 *disruption allele, whereas in this study we characterized an *rfx1 *deletion allele. We do not know why Ty1 cDNA levels dropped precipitously in the *rfx1Δ *strain at 26°C, as Ty1 protein production and processing are not defective at this temperature. If, as suggested by Curcio *et al*., the rate limiting step for pGTy1 elements is integration of cDNA, and Ty1 mobility is temperature sensitive, then perhaps much less Ty1 cDNA is required at lower temperatures to yield His-positive mobility events [31]. This possibility could potentially explain the faint pGTy1 cDNA signal (Figure 6B) and why we were unable to readily detect pGTy1 cDNA using a *HIS3 *probe (data not shown) unlike our previous work in GRF167-derived strains [8]. Additionally, a fortuitous Ty1 transcription termination signal in the *HIS3 *promoter (on the anti-sense strand relative to Ty) causes premature termination in ~50% of transcripts (Curcio J, personal communication). Strain differences in efficiency of transcript termination could also affect levels of pGTy1 cDNA. Given the variability in pGTy1 mobility and cDNA levels between different strain backgrounds, it is perhaps not so unexpected that the increase in pGTy1 mobility in *rfx1Δ *and *sml1Δ *mutants is strain specific. Other host gene mutations affecting Ty1 mobility that demonstrate strain specificity have been described [8].

At permissive temperatures, the primary Ty1 mobility mechanism is mediated by integrase, but at high temperature (34°C), homologous recombination becomes the primary mechanism for genomic integration of Ty1 cDNA [8]. Deletion of *RAD52*, which is required for homologous recombination, reduces or eliminates high temperature mobility in the mutant strains. Given that high temperature mobility is primarily mediated by HR, it seemed plausible that elevated dNTP levels in the mutant strains could mediate an increase in HR of Ty1 cDNA. We conducted a modified gap-repair assay in wild type, *rfx1Δ *and *sml1Δ *strains [8]. n the two deletion strains there was indeed an increase in efficiency of homologous recombination at 34°C compared with wild type. The phenotype was particularly notable in the *rfx1Δ *strain at both 30 and 34°C (Figure 7). This result is consistent with the observation that the *rfx1Δ*-mediated increase in pGTy1 mobility at high temperature is exclusively dependent on HR, as introduction of the *rad52 *mutation eliminated the high temperature mobility phenotype (Figure 5). A recent study reported a novel function of *RFX1 (CRT1) *as a regulator of a Rad52-independent mitotic recombination pathway in yeast [44]. *rad52 rfx1 *double mutants were found to have a spontaneous recombination rate threefold higher than that of a *rad52 *single mutant. However, in contrast to this study, we found that deletion of *rfx1 *alone did not affect recombination rates. The *sml1Δ rad52 *strain retained pGTy1 mobility at high temperature (34°C), suggesting that deletion of *sml1 *increases the efficiency of integrase-mediated transposition events at high temperature by an unknown mechanism. From these recombination experiments, we conclude that the increase in pGTy1 mobility at high temperature in the *rfx1Δ *deletion strain is due to increased HR of available Ty1cDNA, mediated by an increase in cellular dNTP levels.

The shift from integrase-mediated to HR-mediated Ty1 mobility as temperature increases meshes nicely with deregulation of the RNR pathway in *rfx1Δ *and *sml1Δ *strains (Figure 1), because HR-mediated mobility would be expected to require more dNTPs than integrase-mediated mobility. Eukaryotic cells have two recombination pathways for dealing with free DNA ends, namely HR and non-homologous end-joining (NHEJ). The DNA synthesis step in homologous recombination is an energetic and resource-dependent process, requiring high stores of dNTPs to make the process more favourable [45]. Burkhalter *et al*. showed that the induction of RNR in mammalian cell lines increases the use of homologous recombination for chromosomal break repair [45]. Yeast cells innately show a strong preference for HR-mediated rather than NHEJ-mediated recombination [46]. When Ty1 integrase function is inhibited at high temperature, yeast cells resolve free Ty1 cDNA ends through genomic homologous recombination. We found that this HR process is more efficient with increased dNTPs.

### Integration of Ty1 mobility into the S-phase checkpoint pathway

If the increase in pGTy1 mobility at high temperature (34°C) in the *rfx1Δ *and *sml1Δ *strains is the direct result of increased cellular dNTP levels, we would expect to see the high temperature phenotype abolished when cellular dNTP concentrations are lowered. Thus, we assayed pGTy1 mobility in the presence of HU, which inhibits RNR activity and reduces cellular dNTP levels [42,47]. Levels of dNTPs vary during the cell cycle, peaking shortly after the G_1_/S transition [19,48], and treatment of yeast cells with HU reduces dNTP levels to ~80% of G_1 _levels [42]. We found that the high temperature pGTy1 mobility phenotype was eliminated by HU treatment (Figure 8B), and furthermore, mobility frequency was greatly reduced in all strains, even at the lowest levels of HU. Attempts to quantify pGTy1 mobility levels at high temperature (34°C) in the presence of HU were unsuccessful because of the very low levels of mobility. However, pGTy1 mobility was quantified at permissive temperature, and found to be modestly induced by HU treatment (Figure 8A). This result is in agreement with previously published data, showing that HU treatment induced Ty1 mobility and cDNA levels through induction of the S-phase replication stress pathway (Figure 1) [31]. HU induction of Ty1 mobility is independent of *rad9, *and thus results from stalled replication forks resulting from inadequate dNTP levels [31].

In the presence of HU, deletion of the RNR regulators may increase basal dNTP levels to an adequate level to prevent replication fork pausing and subsequent induction of Ty1 mobility through the S-phase replication stress pathway. We found that although pGTy1 mobility was induced in both the wild type and *sml1Δ *strains, mobility was not induced in the *rfx1Δ *strain (Figure 8). In a recent study of the induction of the DNA damage checkpoint, Tang *et al*. carefully quantified levels of all four dNTPs in wild type, *sml1Δ *and *rfx1Δ *(*crt1Δ*) strains during logarithmic growth [16]. The levels of dNTPs in the *rfx1Δ *strain were shown to be modestly but significantly higher than those in an *sml1Δ *strain. Given the tight regulation of cellular dNTP levels, this small difference may be enough to trigger the S-phase replication stress pathway, and subsequently pGTy1 mobility, in the *sml1Δ *strain but not the *rfx1Δ *strain. Activation of the S-phase checkpoint pathways has been directly assayed by measuring the induction of phosphorylated Rad53 protein; however, our laboratory has been unsuccessful in detection of phosphorylated Rad53 [16,49,50].

To further investigate the link of the high temperature mobility phenotype to the S-phase checkpoint pathway, we investigated the effects of manipulating the expression of the upstream kinase Dun1. Activation of Dun1 kinase activity inactivates both Rfx1 and Sml1 (Figure 1). Thus, overexpression of Dun1 might increase dNTP levels and subsequently pGTy1 mobility in wild type strains; however, we found was no effect. Because the plasmid contains the *DUN1 *gene promoter, it is possible that *DUN1 *expression levels from the plasmid are kept at basal levels. Conversely, deletion of *dun1 *would result in more actively repressive forms of Rfx1 and Sml1, and in fact, the levels of cellular dNTPs were found to be reduced in a *dun1Δ *strain [17]. We found that deletion of Dun1 provided a consistent decrease in pGTy1 mobility from 26 to 32°C (Figure 9). This decrease in mobility in the *dun1Δ *strain confirms that Ty1 mobility, both integration and HR mediated, is linked to S-phase checkpoint regulation [31]. Overall, these results support our conclusion that pGTy1 mobility is intimately linked to dNTP levels in the cell and integrated with S-phase checkpoint pathways through regulation of dNTP levels.

## Conclusions

In this study, we found that an increase in dNTP levels through deletion of the RNR regulators Rfx1 and Sml1 stimulated pGTy1 mobility at high temperature (34°C). This increase in pGTy1 mobility at high temperature is not due to an increase of Ty1 cDNA, but rather is mediated by an increase in the efficiency of homologous recombination of existing Ty1 cDNA. These results confirm that at high temperature, when Ty1 protein processing is inhibited, Ty1 mobility is mediated by homologous recombination. Levels of cellular dNTPs, as regulated by components of the S-phase checkpoint pathway, are a limiting factor in HR-mediated Ty1 mobility.

## Methods

### Yeast strains and plasmids

The yeast strains and plasmids used in this study are given in Table 2. Yeast strains were maintained according to standard methods and media prepared as previously described [51]. Plasmid pGTy1H3m*his3*AI, used for the initial genetic screen and quantitative assays, contains a galactose-inducible Ty1 element on a URA3, 2-μm plasmid [35]. Subsequent mobility assays used plasmid pGTy1*his3*AI[*Δ*1] (kind gift of D Garfinkel) [7]. In this plasmid construct, the AI insertion in the plasmid *his3 *marker is located within the corresponding sequence deleted in the *his3Δ1 *marker in strain Hansen BY4741; this eliminates the formation of background His-positive events by homologous recombination of the genomic *his3Δ1 *marker with plasmid sequences. The *DUN1 LEU2*, 2-micron plasmid (pJK679) was made by TOPO TA cloning of a 1948 bp PCR fragment containing the *DUN1 *gene into plasmid pCR2.1-TOPO (Invitrogen, Carlsbad, CA, USA) using the primers JK323 and JK324 (Table 3). The *Xho*I/*Spe*I fragment of this construct was then cloned into the same sites of plasmid pRS425.

**Table 2:** Yeast strains used in this study

JKc number	Parent strain	Genotype	Plasmid name	Source
JKc1046	Hansen BY4741	*MATa his3Δ1 leu2Δ0 met15Δ0 ura3Δ0*	none	Invitrogen

JKc1356	Hansen BY4741	*MATa his3Δ1 leu2Δ0 met15Δ0 ura3Δ0*	pGTy1*his3*AI[*Δ*1] *(URA3, 2 μ*)	Invitrogen

JKc1357	Hansen BY4741	*MATa his3Δ1 leu2Δ0 met15Δ0 ura3Δ0 sml1::kanmx4*	pGTy1*his3*AI[*Δ*1] *(URA3, 2 μ*)	Invitrogen

JKc1358	Hansen BY4741	*MATa his3Δ1 leu2Δ0 met15Δ0 ura3Δ0 rfx1::kanmx4*	pGTy1*his3*AI[*Δ*1] *(URA3, 2 μ*)	Invitrogen

JKc1359	Hansen BY4741	*MATa his3Δ1 leu2Δ0 met15Δ0 ura3Δ0 grh1::kanmx4*	pGTy1*his3*AI[*Δ*1] *(URA3, 2 μ*)	Invitrogen

JKc1360	Hansen BY4741	*MATa his3Δ1 leu2Δ0 ura3Δ0 met15Δ0 rfx1::kanmx4 rad52::LEU2*	pGTy1*his3*AI[*Δ*1] *(URA3, 2 μ*)	This study

JKc1361	Hansen BY4741	*MATa his3Δ1 leu2Δ0 ura3Δ0 met15Δ0 sml1::kanmx4 rad52::LEU2*	pGTy1*his3*AI[*Δ*1] *(URA3, 2 μ*)	This study

JKc1363	Hansen BY4741	*MATa his3Δ1 leu2Δ0 ura3Δ0 met15Δ0 rad52::LEU2*	pGTy1*his3*AI[*Δ*1] *(URA3, 2 μ*)	This study

JKc1424	Hansen BY4741	*MATa his3Δ1 leu2Δ0 met15Δ0 ura3Δ0 sml1::kanmx4 rfx1::LEU2*	pGTy1*his3*AI[*Δ*1] *(URA3, 2 μ*)	This study

JKc1425	Hansen BY4741	*MATa his3Δ1 leu2Δ0 met15Δ0 ura3Δ0 grh1::LEU2*	pGTy1*his3*AI[*Δ*1] *(URA3, 2 μ)*	This study

JKc1426	Hansen BY4741	*MATa his3Δ1 leu2Δ0 met15Δ0 ura3Δ0 grh1::kanmx4 sml1:LEU2*	pGTy1*his3*AI[*Δ*1] *(URA3, 2 μ*)	This study

JKc1427	Hansen BY4741	*MATa his3Δ1 leu2Δ0 met15Δ0 ura3Δ0 rfx1::LEU2*	pGTy1*his3*AI[*Δ*1] *(URA3, 2 μ*)	This study

JKc1428	Hansen BY4741	*MATa his3Δ1 leu2Δ0 met15Δ0 ura3Δ0 rfx1::kanmx4 grh1::LEU2*	pGTy1*his3*AI[*Δ*1] *(URA3, 2 μ)*	This study

JKc1429	Hansen BY4741	*MATa his3Δ1 leu2Δ0 ura3Δ0 met15Δ0 sml1::LEU2*	pGTy1*his3*AI[*Δ*1] *(URA3, 2 μ*)	This study

JKc1445	Hansen BY4741	*MATa his3Δ1 leu2Δ0 met15Δ0 ura3Δ0 dun1::kanmx4*	pGTy1*his3*AI[*Δ*1] *(URA3, 2 μ)*	Invitrogen

JKc1499	Hansen BY4741	*MATa his3Δ1 leu2Δ0 met15Δ0 ura3Δ0*	pGTy1*his3*AI[*Δ*1] *(URA3, 2 μ *pJK679 (*DUN1 LEU2 2 μ)*	This study

JKc1532	Hansen BY4741	*MATa his3Δ1 leu2Δ0 met15Δ0 ura3Δ0 rfx1::LEU2*	None	This study

JKc1533	Hansen BY4741	*MATa his3Δ1 leu2Δ0 ura3Δ0 met15Δ0 sml1::LEU2*	None	This study

**Table 3:** Primers used in the experiments

Primer name	Sequence 5'→3'
U1	GATGTCCACGAGGTCTCT

D1	CGGTGTCGGTCTCGTAG

JK181	TGCGATCTCTTTAAAGGGTG

JK182	TTTGGTGGAGGGAACATCGTT

JK196	CAGATGCGAAGTTAAGTG

JK197	GACAGTCACATCATGCCC

JK198	GGCGATTTGGGAAAAAGTTGAAAAAAAAAATAGCAGTAAAGATTGTACTGAGAGTGCAC

JK199	GTTATATTCTTTTTTAAATATCCCCATATACTAATGATAGCTGTGCGGTATTTCACACCG

JK293	CTTATCTGCTCCTTTGTGATCTTACGGTCTCACTAACCTCAGATTGTACTGAGAGTGCAC

JK294	TAGTAGGACGAGAGTCCCTGAAAAGAAGGGTATCTAAGAGCTGTGCGGTATTTCACACCG

JK295	CACAGCGTGCGAAACTAGGGAAGTAAAAAGGTATAGGAAGAGATTGTACTGAGAGTGCAC

JK296	CCAACTATGCTTTACGTGTTTTGAAGGGAAAGCAAGCTTACTGTGCGGTATTTCACACCG

JK323	AAAAACGATAGGGTGGCACA

JK324	GAAGCCCCTGAATACCATAAA

JK349	AGATGCAATACGACACCAAGA

JK350	CGGAAGAGGTTTTGTCATCA

### Deletion pool transformation, screening and gene identification

A MATa pool of the *S. cerevisiae *deletion library, containing ~5200 unique deleted ORFs, was transformed with plasmid pGTy1H3*mhis3*AI [35]. Transformants (>25,000) were selected on synthetic complete medium lacking uracil (SC-Ura) for 3 days at 30°C. Colonies were replica plated to galactose medium lacking uracil prewarmed to 34°C, and incubated for 48 hours. Cells were then replica plated to SC-His prewarmed to 34°C. Colonies showing greater growth than the wild type on SC-His were patched from the SC-Ura transformation plates to fresh medium, and assayed to confirm the high temperature mobility phenotype. The deleted ORF was identified from nine independent clones, each showing high temperature pGTy1 mobility. Genomic DNA was isolated from positive clones and used in a PCR reaction employing the common primers U1 and D1 (Table 3). Three clones did not amplify a product using the U1 and D1 primers. For these clones, primers complementary to the *kanmx *deletion gene and heading towards the downtag (JK196) or the uptag (JK197) sequences were paired with primers D1 or U1 respectively. A product was obtained using the U1/uptag primer pair. The resulting PCR products were purified (PCR Purification Kit; Qiagen, Valencia, CA, USA) and sequenced using the uptag primer. The resulting sequence was compared with the deletion uptag and downtag sequences to identify the disrupted ORF.

### Ty1 mobility assays

Patch assays and quantitative assays were performed as previously described [8]. For the HU assays, patches were grown on SC-Ura plates for 24 to 36 hours at 30°C and then replica plated to SC-Ura with 2% galactose and varying concentrations of HU (0 to 100 mmol/l). Plates were equilibrated to the induction temperature before replica plating. After 24 hours, patches were replica plated to SC-His at 30°C. Papillae are usually visible within 24-36 hours.

For quantification, strains were grown as 12 × 12 mm patches on SC-Ura plates at 30°C for 24 to 36 hours. The patches were then transferred into 15 mL of SC-Ura plus 2% galactose medium with varying concentrations of HU with an optical density at 600 nm (OD_600_) of approximately 0.4. The medium was pre-incubated to the induction temperature. After 2 days of growth, the OD_600 _was approximately 1.2. A counting chamber (Number 100 Hy-Lite Chamber; Hausser Scientific, Horsham, PA, USA) was used to count cells, giving a volume ~ 0.5-1 × 10^8 ^cells/ml. Cells were then diluted and plated onto SC-His to assess frequency of pGTy1 mobility and to yeast-extract peptone dextrose (YPD) agar to determine the number of viable cells. For assessing the frequency of pGTy1 mobility at 28°C, 100 μl of cells at ~1000 cells/μl were plated onto SC-His. At 34°C mobility is much lower, so that 0.5-1 ml of cells were plated onto SC-His. For assessing viability, cells were diluted to ~1 cell/μl and 100 μl was plated to YPD. Frequency of pGTy1 mobility was calculated as the number of His-positive papillae/total cells plated. All strains were patched, inoculated and plated in triplicate for each temperature and HU concentration. The average mobility and standard deviation was calculated for each point.

### Rad52 strain construction

Plasmid pSM20, containing the *rad52::LEU2 *allele, was digested with *Bam*HI. Digestion was confirmed by gel electrophoresis, yielding two bands of 5 kbp and 4.6 kbp. The remaining digest was transformed into yeast [52], and cells were plated to SC-Leu medium to select for integration of the disrupted *rad52 *allele. Isolated transformants were confirmed by ultraviolet-induced growth sensitivity compared with the isogenic *RAD52 *parent.

### Deletion constructs

PCR products for targeted deletions of ORFs with *kanmx *or *LEU2 *was obtained by using pRS400 or pRS425, respectively, as target DNA as described previously [53]. For *RFX1 *deletion constructs, the forward primer JK198 and the reverse primer JK199 were used; or *SML1 *deletion constructs, the forward primer JK293 and the reverse primer JK294 were used; and for *GRH1 *deletion constructs, the forward primer JK295 and reverse primer JK296 were used. PCR conditions were as described previously, using a thermostable polymerase (PfuTurbo; Stratagene, a division of Agilent, Santa Clara, CA, USA). The resulting PCR product was transformed into competent yeast strains by lithium acetate transformation [52].

### Immunoblotting

Cell growth and protein isolation were performed as described previously [8]. Whole cell extracts were mixed with an equal volume of 2 × sample loading buffer (20% v/v glycerol, 0.125 M Tris-HCl, pH 6.8, 5% w/v SDS, 10% v/v 14 mol/l β-mercaptoethanol, 0.2% w/v bromophenol blue) and boiled for 3 minutes before loading onto a 10% (Gag blots) or 7.5% (Pol blots) SDS gel. Gels were transferred to nitrocellulose (Gag blots) or PVDF (Pol blots) membranes in Tris-glycine buffer containing 10% methanol at 24 V for 1 hour. Membranes were blocked in buffer I (15 mM Tris, pH8, 150 mmol/l NaCl, 0.25% w/v gelatine (G7041, Sigma Chemical Co, St Louis, MO, USA) and 0.25%v/v Tween-20) overnight at 4°C. Membranes were then probed with antibody for 1 hour at room temperature in Buffer II (15 mmol/l Tris, pH8, 150 mmol/l NaCl, 0.25% w/v gelatine, 0.25%v/v Tween-20 and 7.5 mmol/l EDTA) and washed twice for 5 minutes in fresh Buffer II. The appropriate secondary antibody was diluted in Buffer II and added to the membrane, which was then washed four times for 5 minutes each in Buffer II, followed by Amersham ECL (nitrocellulose) or Amersham ECL-Plus (PVDF) reagent (GEHealthcare Life Sciences, Piscataway, NJ USA) and exposed on a image station (2000R; Eastman Kodak Co., Rochester, NY, USA). Anti-Gag (anti-VLP polyclonal serum R2-F) and anti-integrase (8B-11 monoclonal antibody) were as described previously [54,55]. Rabbit polyclonal antiserum to a synthetic peptide (CTKYTPDKKIVSNAS) of the yeast protein Gadph (*TDH1*),was used as a loading control.

### cDNA Southern analysis

For induction of Ty1 cDNA, cells were inoculated into 100 ml VLP medium plus raffinose at a starting OD_600 _of ~0.15, and grown at 30°C for 4 hours. Galactose was added to a final concentration of 2%, and cells were grown for 12 hours at the induction temperature. Genomic DNA was isolated as described previously [56]. Aliquots (10 μl; ~ 50-100 μg) of DNA were digested with 5 U *Sph*I for 20 hours in a 50 μl reaction containing 1 μl RNase (10 mg/ml). Samples were electrophoretically separated (0.75% agarose gel) and transferred to a hybridization transfer membrane (GeneScreen; Perkin Elmer, Fremont, CA, USA) and hybridized to a 887 bp probe. The probe was generated using pGTy1H3m*his3*AI*Δ*1 as template DNA in a PCR reaction with 25 cycles of denaturing at 95°C for 1 minute, annealing at 59°C for 1 minute and extension at 72°C extension for 1 minute) with primers JK349 and JK35 (Table 3). The PCR product (5 μl) was labelled (NEBlot Kit; New England Biolabs, Ipswich, MA, USA), purified (Nucleotide Removal Kit; Qiagen) and hybridized to genomic DNA as described previously [8]. The location of expected bands was determined from a standard curve generated using the molecular weight standards. The membrane was washed twice for 5 minutes in 2× saline sodium citrate buffer at room temperature and exposed overnight at -80°C to film with an enhancing screen. For quantifying the signal, the image file of the scanned film was imported into image analysis software (1D; Kodak). The image lane and band location functions, and the net intensity, which is the sum of the background-subtracted pixel values in the band rectangle, were used to determine the ratios of the relative amounts of cDNA in each band.

### Recombination assays

Strains JKc1046, JKc1532 and JKc1533 were inoculated into YPD at 30 or 34°C at an absorbance at 600 nm (A_600_) of ~0.05 and grown to an A_600 _of ~0.7. Cells were washed twice in water and frozen at -80°C in 0.1 M lithium acetate/Tris EDTA buffer with 15% glycerol. The *HIS3 *linear DNA fragment for transformation was made using plasmid pJK592 (0.2 ng/μL) as template DNA with primers JK181 and JK182 (Table 3), generating a 590 bp product. Cell aliquots (100 μl) were thawed and transformed with 7.8 μg of PCR product as described previously [52], except that for 34°C transformation reactions, reagents were preheated to 34°C and all incubation steps were carried out at 34°C. Plasmid pJK592 was transformed (0.7 μg into JKc1532; 0.07 μg into JKc1046 and JKc1533) to assess efficiency of transformation. His-positive prototrophs were selected by plating onto SC-His medium at 30 or 34°C. Recombination efficiency was determined from transformation of the PCR product and was calculated as number of colony-forming units (cfu) per total number of cells in the transformation reaction as determined by direct cell counts in a thawed aliquot. The recombination efficiency was normalized to wild type, and then multiplied by a transformation efficiency factor, the inverse of the average number of His-positive cfu/total cell number normalized to wild type for transformation of plasmid DNA. Each strain was transformed in triplicate at both temperatures. Results are reported as the means and standard deviation for each set.

## Competing interests

The authors declare that they have no competing interests.

## Authors' contributions

JO performed the Southern blot analyses, HU assays, *DUN1 *cloning and deletion construction and assays, and drafted the introduction, parts of the results and the conclusions. MG created the deletion strains and *rad52 *constructs, and performed the mobility assays and homologous recombination assays, and also attempted to develop an *in vitro *assay to measure reverse transcriptase activity at varying dNTP concentrations (data not shown). JK supervised the research, helped with the quantitative assays, performed the protein analysis and finalized the manuscript.

## Authors' information

MG participated in this study as an undergraduate student at Juniata College. receiving a BS in May 2009, and is currently enrolled in the graduate program at University of Kentucky. JO completed work on the project as an undergraduate at Juniata College, and it formed the basis of his undergraduate honors thesis. He received his BS in May 2010 and is currently enrolled in the graduate program at Cornell University. JK is the David K. Goodman '74 Endowed Chair in Biology at Juniata College.

## Supplementary Material

Additional file 1 **Figure 2 data**. Numerical values for data shown in Figure 2. A table of the average (+/- standard deviation) values of His-positive prototroph formation for each of the points graphed in Figure 2. Click here for file

Additional file 2 **Figure 5 data**. Numerical values for data shown in Figure 5. A table of the average (+/- standard deviation) values of His-positive prototroph formation for each of the points graphed in Figure 5. Click here for file

Additional file 3 **Figure 8A data**. Numerical values for data shown in Figure 8A. A table of the average (+/- standard deviation) values of His-positive prototroph formation for each of the points graphed in Figure 8A. Click here for file
