# Supplementary material for: Regulators of ribonucleotide reductase inhibit Ty1 mobility in saccharomyces cerevisiae
Source: Mob DNA. 2010 Nov 22;1:23. doi: 10.1186/1759-8753-1-23 (PMC3002893; doi:10.1186/1759-8753-1-23)
Supplement: Additional file 1 — Figure 2 data. Numerical values for data shown in Figure 2. A table of the average (+/- standard deviation) values of His-positive prototroph formation for each of the points graphed in Figure 2. [file 1759-8753-1-23-S1.PDF]

Numerical values of frequency of His+ prototroph formation for data shown in figure 2.

Strain	Relevant genotype	Temperature of galactose induction	Frequency of His+ prototrophs/cells plated (+/- S.D.)
JKc1356	wild type	28	49 (+/- 17) x 10^{-5}[a]
JKc1358	*rfx1Δ*	28	77 (+/- 9.7) x 10^{-5}
JKc1357	*sml1 Δ*	28	77 (+/- 2.8) x 10^{-5}
JKc1359	*grh1 Δ*	28	88 (+/- 6.1) x 10^{-5}
JKc1356	wild type	30	24 (+/- 0.03) x 10^{-5}
JKc1358	*rfx1Δ*	30	38 (+/- 5.1) x 10^{-5}
JKc1357	*sml1 Δ*	30	58 (+/- 2.1) x 10^{-5}
JKc1359	*grh1 Δ*	30	56 (+/- 2.1) x 10^{-5}
JKc1356	wild type	32	0.98 (+/- 0.22) x 10^{-5}
JKc1358	*rfx1Δ*	32	3.6 (+/- 1.7) x 10^{-5}
JKc1357	*sml1 Δ*	32	5.0 (+/- 0.89) x 10^{-5}
JKc1359	*grh1 Δ*	32	7.4 (+/- 0.62) x 10^{-5}
JKc1356	wild type	34	0.30 (+/- 0.17) x 10^{-5}
JKc1358	*rfx1Δ*	34	1.0 (+/- 0.25) x 10^{-5}
JKc1357	*sml1 Δ*	34	1.2 (+/- 0.46) x 10^{-5}
JKc1359	*grh1 Δ*	34	2.9 (+/- 0.72) x 10^{-5}

[a] Each value indicates the average number of His+ prototrophs per cell from three separate patches (+/- standard deviation)
