# Supplementary material for: Regulators of ribonucleotide reductase inhibit Ty1 mobility in saccharomyces cerevisiae
Source: Mob DNA. 2010 Nov 22;1:23. doi: 10.1186/1759-8753-1-23 (PMC3002893; doi:10.1186/1759-8753-1-23)
Supplement: Additional file 2 — Figure 5 data. Numerical values for data shown in Figure 5. A table of the average (+/- standard deviation) values of His-positive prototroph formation for each of the points graphed in Figure 5. [file 1759-8753-1-23-S2.PDF]

Numerical values of frequency of His+ prototroph formation for data shown in figure 5.

Strain	Relevant genotype	Temperature of galactose induction	Frequency of His+ papillae (+/- S.D.)
JKc1363	*rad52*	28	21 (+/- 3.5) x 10^{-5}[a]
JKc1360	*rad52 rfx1Δ*	28	24 (+/- 3.0) x 10^{-5}
JKc1361	*rad52 sml1 Δ*	28	20 (+/- 0.10) x 10^{-5}
JKc1363	*rad52*	30	3.8 (+/- 2.0) x 10^{-5}
JKc1360	*rad52 rfx1Δ*	30	3.0 (+/- 1.7) x 10^{-5}
JKc1361	*rad52 sml1 Δ*	30	2.0 (+/- 1.2) x 10^{-5}
JKc1363	*rad52*	32	0.01 (+/- .02) x 10^{-5}
JKc1360	*rad52 rfx1Δ*	32	0.18 (+/- 0.13) x 10^{-5}
JKc1361	*rad52 sml1 Δ*	32	0.49 (+/- 0.09) x 10^{-5}
JKc1363	*rad52*	34	0.11 (+/- 0.02) x 10^{-5}
JKc1360	*rad52 rfx1Δ*	34	0.13 (+/- 0.04) x 10^{-5}
JKc1361	*rad52 sml1 Δ*	34	0.59 (+/- 0.17) x 10^{-5}

[a] Each value indicates the average number of His+ prototrophs per cell from three separate patches (+/- standard deviation)
