# Supplementary material for: Regulators of ribonucleotide reductase inhibit Ty1 mobility in saccharomyces cerevisiae
Source: Mob DNA. 2010 Nov 22;1:23. doi: 10.1186/1759-8753-1-23 (PMC3002893; doi:10.1186/1759-8753-1-23)
Supplement: Additional file 3 — Figure 8A data. Numerical values for data shown in Figure 8A. A table of the average (+/- standard deviation) values of His-positive prototroph formation for each of the points graphed in Figure 8A. [file 1759-8753-1-23-S3.PDF]

Numerical values of frequency of His+ prototroph formation for data shown in figure 8A.

strain	Relevant genotype	0 mM HU	25 mM HU	50 mM HU	75 mM HU	100 mM HU
JKc1356	wild type	68 (+/-30) x 10^{-5}[a]	240 (+/-65) x 10^{-5}	330 (+/-46) x 10^{-5}	280 (+/-19) x 10^{-5}	400 (+/- 43) x 10^{-5}
JKc1358	rfx1Δ	320 (+/-19) x 10^{-5}	150 (+/-16) x 10^{-5}	180 (+/-56) x 10^{-5}	46 (+/-9.1) x 10^{-5}	220 (+/- 62) x 10^{-5}
JKc1357	sml1 Δ	190 (+/-23) x 10^{-5}	360 (+/-100) x 10^{-5}	300 (+/-42) x 10^{-5}	280 (+/-16) x 10^{-5}	360 (+/- 40) x 10^{-5}

[a] Each value indicates the average number of His+ prototrophs per cell from three separate cultures (+/- standard deviation)
